# Temporal Trends in Physician Burnout During and After the COVID‐19 Pandemic in Japan: A Repeated Cross‐Sectional Study

**DOI:** 10.1002/jgf2.70101

**Published:** 2026-02-05

**Authors:** Akira Kuriyama, Kiyoshi Shikino

**Affiliations:** ^1^ Department of Health Research Methods, Evidence, and Impact McMaster University Hamilton Ontario Canada; ^2^ Department of Community‐Oriented Medical Education Chiba University Graduate School of Medicine Chiba Japan; ^3^ Department of General Medicine Chiba University Hospital Chiba Japan

**Keywords:** COVID‐19 pandemic, internist, physician burnout, primary care physician, repeated cross‐sectional study

## Abstract

**Background:**

To examine how burnout among Japanese internists and primary care physicians evolved from the onset of the coronavirus disease‐2019 (COVID‐19) pandemic to the post‐pandemic period.

**Methods:**

We reanalyzed data from five web‐based surveys of members of the American College of Physicians—Japan Chapter between January 2020 and April 2024. Burnout was assessed using the Japanese version of the Mini‐Z 2.0 survey. The survey dates corresponded to major pandemic phases: early pandemic (January 2020), first‐wave peak (June 2020), mid‐pandemic (March 2021), post‐emergency (December 2022), and recovery phase (April 2024). Temporal trends in burnout prevalence were analyzed using the Cochran–Armitage test.

**Results:**

A total of 1099 respondents were included. Burnout prevalence was the highest during the early pandemic (34.6% in January 2020, 34.5% in June 2020), declined modestly during the mid‐pandemic phase (31.8% in March 2021), and further decreased to 26.2% by April 2024. A significant linear decline in burnout prevalence was observed over time (*P*trend = 0.038). Reduction in burnout corresponded with the easing of pandemic restrictions, vaccine rollout, and stabilization of healthcare operations.

**Conclusions:**

Burnout among Japanese internists and primary care physicians declined significantly from the peak of the COVID‐19 pandemic to the recovery phase. However, approximately one‐quarter of physicians remained affected in 2024.

## 1 Introduction

The coronavirus disease 2019 (COVID‐19) pandemic placed unprecedented stress on healthcare systems worldwide and triggered a surge in physician burnout and mental health problems [1, 2]. Physician burnout, characterized by emotional exhaustion, depersonalization, and reduced personal accomplishment [3], is associated with depression [4], suicidal ideation [5], and diminished patient safety [6]. During the pandemic, surveys reported burnout in approximately one‐third to more than half of healthcare professionals globally [7]. Such high burnout prevalence threatens both patient care quality and workforce stability. As the acute crisis subsides, whether burnout represents a transient reaction or a lasting consequence of the pandemic is still unclear [8].

In Japan, the government implemented strict public health measures, including multiple state emergency declarations, to control viral spread [9]. These measures coincided with increased workload and administrative burden on clinicians [10]. Early studies in Japan documented considerable distress among healthcare workers [11, 12], and approximately one‐third of Japanese internists and primary care physicians reported burnout symptoms during the first wave of COVID‐19 [13]. However, the majority of such studies in Japan and globally have focused on single‐phase cross‐sectional surveys, providing little insight about how burnout prevalence evolved as the pandemic progressed and eventually subsided.

To address this gap, we conducted a multiyear observational study, using a repeated cross‐sectional design, to track temporal trends in physician burnout in Japan from the pandemic onset to the post‐pandemic phase in Japan (2020–2024). In addition to documenting the changes in burnout prevalence across multiple phases, we also interpreted these findings in the context of Japanese social and system factors, which may provide insights for supporting physician well‐being in future public health crises.

## 2 Methods

### 2.1 Study Design and Participants

We conducted a repeated cross‐sectional study using five web‐based cross‐sectional surveys to assess physician burnout in Japan. Surveys were administered to members of the American College of Physicians—Japan Chapter, representing internists and primary care physicians practicing in Japan. For each survey, an email invitation with a survey link was distributed via the association's mailing listserv, employing a convenience sampling approach [14] to invite all members except for student members. Participation was voluntary, and informed consent was obtained electronically.

All surveys collected the following information: participant demographics (sex and years of clinical experience) and physician burnout, which was assessed using the Mini‐Z 2.0 Burnout Survey [13, 15]. The instrument for measuring burnout included a single‐item measure, and respondents rated their burnout level on a five‐point Likert scale, where “5” indicates no burnout symptoms and “1” indicates severe burnout [13, 15]. Burnout was defined as a score of ≤ 3 on the scale in accordance with the original scoring direction and definition of burnout in the Mini‐Z 2.0 instrument. This tool was validated for measuring professional healthcare burnout and work conditions [13, 15] and has been used extensively. The Japanese version of the Mini‐Z 2.0, translated and culturally adapted, was consistently used across all surveys [13]. Each survey questionnaire was pilot‐tested with a small group of physicians to ensure clarity and technical functionality, and a minor wording adjustment was made before final deployment.

Surveys were conducted at the following time points, corresponding to key phases of the COVID‐19 pandemic in Japan: early pandemic (January 2020), peak of the first wave (June 2020), mid‐pandemic when the fourth wave emerged shortly after the third (March 2021), low severity but high caseload surge period (December 2022), and post‐pandemic recovery (April 2024) [11, 12, 13, 16, 17]. These survey periods were predetermined to capture the representative phases of the pandemic based on Japan's national COVID‐19 epidemiology and policy milestones.

### 2.2 Statistical Analysis

Our primary outcome was the prevalence of burnout at each survey. Because participants were not necessarily consistent across surveys, each survey was treated as an independent cross section, and the overall pattern was interpreted as a temporal ecological trend. We used the Cochran–Armitage test to assess the trend in burnout prevalence between June 2020 and April 2024. We hypothesized a linear downward trend in burnout prevalence over time. The January 2020 data were treated as pre‐pandemic baseline data reflecting the period immediately after COVID‐19 emergence; however, because this time point preceded major system‐level stress and the first state‐of‐emergency declaration, it was not included in the trend test. Statistical significance was set at a two‐tailed *p*‐value < 0.05 for trend. All analyses were conducted using Stata (version 17; StataCorp, College Station, TX, USA). We descriptively interpreted temporal changes in burnout in the context of major external events (e.g., state‐of‐emergency periods), although we acknowledge that such associations are not necessarily causal.

We utilized previously published data, and ethical approval for each survey was obtained, as detailed in the original publications [11, 12, 13, 16, 17].

## 3 Results

### 3.1 Participant Characteristics

Across the five surveys, 1099 physicians responded (283, 322, 214, 177, and 103 in chronological order) (Table 1). The proportion of female physicians ranged from 11.2% to 19.8%, without a significant change over time. The distribution of clinical experience shifted slightly toward more senior physicians: in 2020, 40.6% had > 26 years of experience, which increased to approximately 45% in 2022 and 2024. Approximately half of the respondents practiced in urban settings. No significant differences were found in sex or clinical experience of the respondents (*χ* ^2^ test, *p* = 0.072 and 0.062, respectively), whereas practice location differed significantly (*χ* ^2^ test, *p* = 0.036) between June 2020 and April 2024.

**TABLE 1:** Characteristics of participants from January 2020 to April 2024.

Characteristics	January 2020	June 2020	March 2021	December 2022	April 2024
Participants, *n*	283	322	214	177	103
Response rates, %	22.6	25.9	18.2	15.7	9.7
Female, *n* (%)	39 (13.8)	44 (13.7)	24 (11.2)	35 (19.8)	19 (18.4)
Career duration (years), *n* (%)					
1–5	22 (7.8)	30 (9.3)	13 (6.0)	12 (6.8)	9 (8.8)
6–15	75 (26.5)	47 (14.6)	31 (14.5)	31 (17.5)	17 (16.5)
16–25	71 (25.1)	87 (27.0)	47 (22.0)	31 (17.5)	30 (29.1)
> 26	115 (40.6)	158 (49.1)	123 (57.5)	80 (45.2)	47 (45.6)
Unspecified	0 (0)	0 (0)	0 (0)	22 (12.4)	0 (0)
Practice location, *n* (%)					
Urban	151 (53.3)	160 (49.7)	123 (57.5)	NA	44 (42.7)
Suburban	57 (20.1)	71 (22.0)	52 (24.3)	NA	42 (40.8)
Rural	75 (26.5)	91 (28.3%)	39 (18.2)	NA	17 (16.5)
Burnout, *n* (%)	98 (34.6)	111 (34.5)	68 (31.8)	47 (26.6)	27 (26.2)

*Note:* The practice location was not collected in the December 2022 survey. In 2022, 22 respondents did not report their career duration and were categorized as “Unspecified,” explaining the discrepancy between the total number of participants and the career‐duration subtotals.

Abbreviation: NA, not available.

### 3.2 Burnout Prevalence Over Time

**FIGURE 1:**
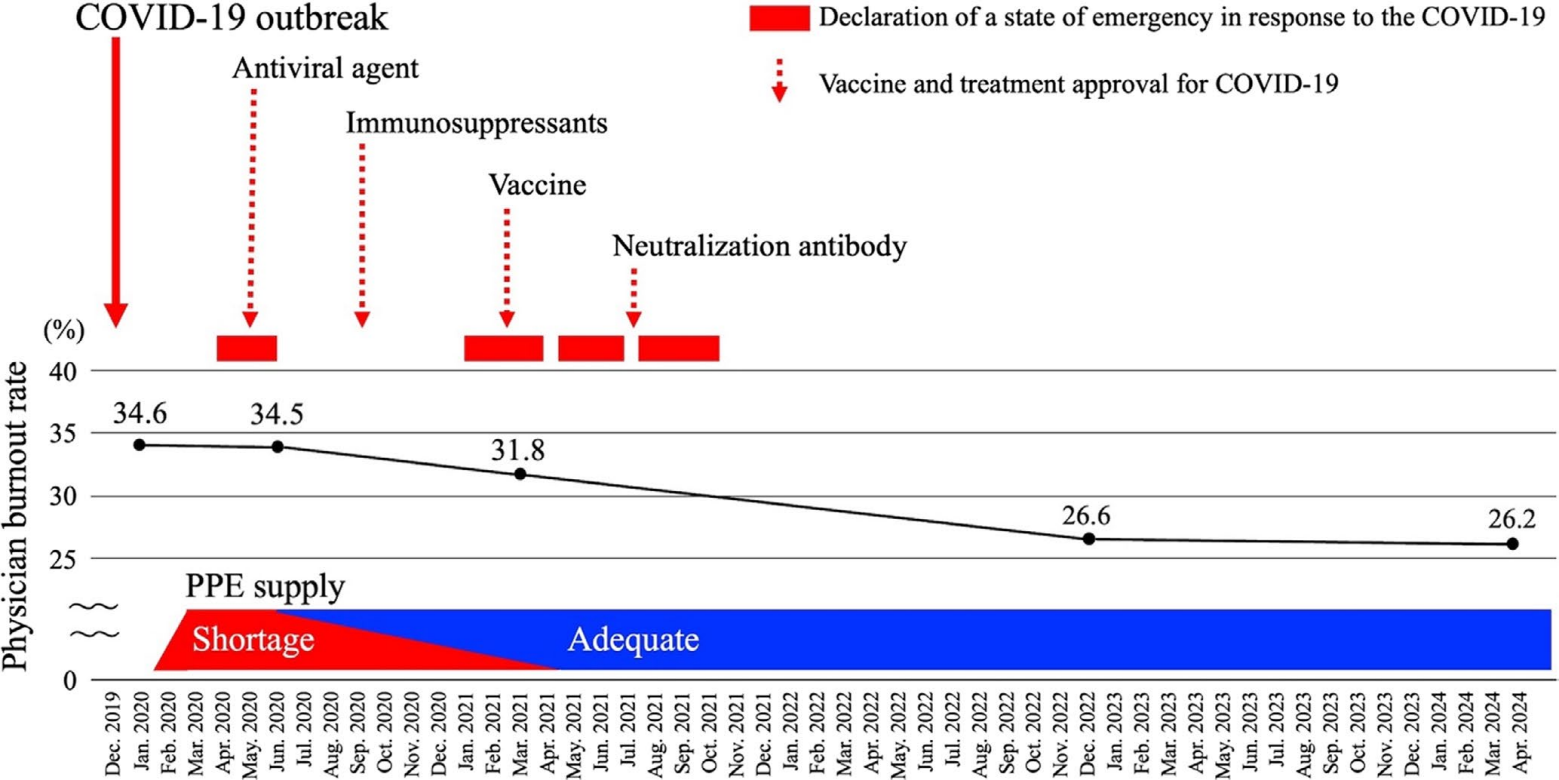
Temporal trends in physician burnout rates from January 2020 to April 2024, highlighting periods of state emergency declarations. COVID‐19, Coronavirus disease 2019; PPE, Personal protective equipment.

The prevalence of burnout showed a clear declining trend from 2020 to 2024 (Figure 1). During the initial phase of the COVID‐19 pandemic, which coincided with Japan's first state‐of‐emergency declaration, the prevalence of burnout was 34.6% in January 2020 (immediately after COVID‐19 emergence) and 34.5% in June 2020 (the peak of the first wave). Burnout gradually declined after mid‐2020, as the immediate crisis pressure eased. In March 2021, at the transition between the third and fourth waves, burnout decreased modestly, and further improvement was observed after the lifting of emergency measures and vaccine rollout. In December 2022, the burnout prevalence fell to 26.6%, reaching 26.2% in April 2024 (the final survey), the lowest level across our surveys.

A significant linear declining trend was observed in the physician burnout prevalence (*P*trend = 0.038). The highest burnout prevalence coincided with peak pandemic stress (e.g., during state‐of‐emergency and high COVID‐19 burden in 2020), and the lowest occurred in the recovery phase when Japan lifted all emergency measures and entered a post‐pandemic state (Figure 1).

## 4 Discussion

In this repeated cross‐sectional study of surveys with Japanese internists and primary care physicians, we observed a significant linear decline in burnout from the peak of the COVID‐19 pandemic to the post‐pandemic period. Burnout affected approximately one‐third of physicians during the acute pandemic phase in 2020, when healthcare providers were under heavy strain. By April 2024, roughly 1 year after Japan downgraded its COVID‐19 legal status and daily life began returning to normal, burnout prevalence declined to approximately 26%.

Previous studies have documented changes in mental health outcomes, including burnout, during the COVID‐19 pandemic. A longitudinal study [18] and meta‐analyses [19] reported an overall increase in burnout among healthcare professionals, compared with pre‐pandemic levels. However, findings from 2020 to 2022 remain inconsistent. For instance, repeated surveys in a Canadian province reported an increase in physician burnout from 29.0% in March 2020 to 34.6% in April 2021 [20]. Similarly, repeated surveys in the United States reported an increase of physicians reporting burnout symptoms from 38.2% in 2020 to 62.8% in 2021 [21]. A longitudinal study using the Maslach Burnout Inventory assessing occupational burnout [22] observed higher burnout among Canadian emergency physicians in September 2022 than in November 2020 [23]. Conversely, a narrative review reported improvements in stress, post‐traumatic stress disorder, and insomnia among healthcare professionals [24]. Our findings, involving Japanese physicians, differ in two respects: burnout did not rise substantially immediately after the pandemic onset, and a significant and sustained decline was observed from mid‐2020 through 2024.

The pattern of burnout observed in our study paralleled key milestones in Japan's pandemic response. Burnout peaked during the first and second waves, marked by a shortage of personal protective equipment (PPE) [25, 26], limited information about COVID‐19, and the absence of disease‐specific vaccines and therapies [27]. These deficits could have heightened physicians' perceived risk, workload, and moral distress, thereby exacerbating burnout during the early pandemic [28, 29]. After the approval of vaccines on February 14, 2021, and therapies such as remdesivir on May 7, 2020, dexamethasone on July 17, 2020, and casirivimab or imdevimab on July 19, 2021, in Japan, burnout levels declined modestly. Moreover, a nationwide supply of PPE was stabilized through expanded domestic production [30, 31], and updated government evidence‐based clinical practice guidelines provided clearer therapeutic algorithms [32]. As frontline physicians accumulated greater hands‐on experience with COVID‐19 care [33], uncertainty and anxiety decreased, likely contributing to the further decline in burnout. These contextual factors may align with the decline in burnout prevalence from June 2020 to April 2024.

The discrepancy in the trajectory of burnout prevalence between Japan and other countries may reflect variations in study design and policy environments. First, our study used a repeated cross‐sectional design rather than a longitudinal one; different individuals may have participated in each survey, and this could have confounded the comparisons across waves. Second, governmental policies varied across countries. For instance, although many countries implemented legally enforced lockdowns, the Japanese government relied on public, voluntary behavioral restrictions and declared a state of emergency without legal penalties [34, 35]. Countries that implemented stringent policies more promptly experienced a significant reduction in the prevalence of depressive symptoms when the restrictions were lifted [36]. Third, the caseload and timing of the pandemic peak differed across countries, complicating cross‐country comparisons. Furthermore, burnout measurements were not synchronized across studies. If burnout had been assessed at shorter intervals, our findings may have differed. Major disruptive events such as pandemics and disasters are rare, and comparable longitudinal evidence remains limited, making it challenging to draw causal inferences. Future research should involve collaboration with both local and national governments to monitor the mental health of essential workers, including healthcare professionals, and track the same cohort longitudinally, preferably through a census, to assess the effects of policy shifts and workload changes on their mental health trajectories. Such systems can enable real‐time assessment of the mental health impact of policy shifts and workload changes and facilitate timely interventions and resource allocation. Our policy proposal should be implemented nationally to enable cross‐national comparisons of how social factors affect healthcare professionals.

Although the burnout prevalence declined over time, it remained substantial in 2024, with one‐quarter of our respondents still affected. A national survey in the United States similarly found that the burnout prevalence in 2023, although lower than the 2022 peak, remained above the 2018 baseline [37]. A systematic review in 2023 also found a post‐pandemic burnout prevalence of 68%, compared with 42% before the pandemic [19]. These findings indicate that physician burnout has not fully returned to pre‐pandemic levels and highlight the need for sustained mitigation strategies. Persistent burnout may reflect factors unrelated to COVID‐19, such as chronic workforce shortages and aging populations with complex needs, as well as the lingering pandemic effects, such as delayed care, personal exhaustion, and bereavement [38]. Although the decline we observed suggests partial recovery, the proportion of physicians experiencing burnout in 2024 remains clinically meaningful. In Japan, structural challenges—including chronic physician shortages, increasing care complexity due to population aging, and regional disparities in healthcare resources—may continue to place a psychological burden on physicians regardless of pandemic conditions. Therefore, governmental monitoring and supportive measures remain necessary to maintain physicians' well‐being and resilience of the healthcare system.

Our study has some strengths. We measured burnout prevalence among internists and primary care physicians across all key phases of the COVID‐19 pandemic in Japan, at five time points, nationwide sampling, and a single validated burnout measurement scale, which enabled consistent comparability. However, this study also has some limitations. First, the survey questionnaire was distributed only to members of a single professional society, which may limit the generalizability. Although the demographic characteristics of the ACP Japan Chapter members (e.g., age distribution, sex, and geographic representation) broadly resemble those of the national physician workforce, they may differ in professional engagement or interest in general internal medicine, and thus, the possibility of selection bias cannot be ignored. Second, the response rate declined over time, potentially introducing non‐response bias. However, emerging evidence indicates that lower response rates do not necessarily bias estimates [39, 40]. Third, minor changes with participant demographics, such as practice location, may have influenced the burnout prevalence. Fourth, although the Mini‐Z 2.0 single‐item burnout measure is validated and widely used, it cannot distinguish specific dimensions of burnout and offers more limited construct coverage than multidimensional scales such as the Maslach Burnout Inventory. Despite these limitations, to our knowledge, this is the only serial assessment of physician burnout in Japan throughout the pandemic. Our findings contribute to the limited global evidence on healthcare professionals' mental health trajectories throughout the pandemic and offer insights for managing future global public health crises.

## 5 Conclusion

This repeated cross‐sectional study of internists and primary care physicians in Japan found a significant decline in burnout from the peak of the COVID‐19 pandemic to its aftermath. Our contextual interpretation may suggest the impact of external stressors on physicians' mental health and the potential for recovery once those stressors subside. However, a considerable proportion of physicians continue to experience burnout in the post‐pandemic period, underscoring the need for sustained attention to work environments and support systems in the post‐pandemic era.

## Author Contributions

**Kiyoshi Shikino:** conceptualization, data curation, investigation, visualization, resources, writing – original draft, writing – review and editing. **Akira Kuriyama:** conceptualization, methodology, software, data curation, investigation, visualization, supervision, writing – original draft, writing – review and editing, validation.

## Funding

The authors have nothing to report.

## Ethics Statement

This study used only publicly available, anonymized data; therefore, ethics committee approval was not required.

## Consent

The authors have nothing to report.

## Conflicts of Interest

The authors declare no conflicts of interest.

## Data Availability

The dataset used and analyzed during this study is available from the corresponding author upon reasonable request.
